# Effect of the Human Amniotic Membrane on Liver Regeneration in Rats

**DOI:** 10.1155/2015/706186

**Published:** 2015-09-17

**Authors:** Mesut Sipahi, Sevinç Şahin, Ergin Arslan, Hasan Börekci, Bayram Metin, Nuh Zafer Cantürk

**Affiliations:** ^1^Department of General Surgery, School of Medicine, Bozok University, 66100 Yozgat, Turkey; ^2^Department of Pathology, School of Medicine, Bozok University, 66100 Yozgat, Turkey; ^3^Department of Thoracic Surgery, School of Medicine, Bozok University, 66100 Yozgat, Turkey; ^4^Department of General Surgery, School of Medicine, Kocaeli University, 41000 Kocaeli, Turkey

## Abstract

*Introduction*. Operations are performed for broader liver surgery indications for a better understanding of hepatic anatomy/physiology and developments in operation technology. Surgery can cure some patients with liver metastasis of some tumors. Nevertheless, postoperative liver failure is the most feared complication causing mortality in patients who have undergone excision of a large liver mass. The human amniotic membrane has regenerative effects. Thus, we investigated the effects of the human amniotic membrane on regeneration of the resected liver.* Methods*. Twenty female Wistar albino rats were divided into control and experimental groups and underwent a 70% hepatectomy. The human amniotic membrane was placed over the residual liver in the experimental group. Relative liver weight, histopathological features, and biochemical parameters were assessed on postoperative day 3.* Results*. Total protein and albumin levels were significantly lower in the experimental group than in the control group. No difference in relative liver weight was observed between the groups. Hepatocyte mitotic count was significantly higher in the experimental group than in the control group. Hepatic steatosis was detected in the experimental group.* Conclusion*. Applying the amniotic membrane to residual liver adversely affected liver regeneration. However, mesenchymal stem cell research has the potential to accelerate liver regeneration investigations.

## 1. Introduction

The liver is a vital metabolic organ and is the first of many locations for the development of cancer metastases due to dual feeding by the hepatic artery and portal vein. Hepatic resection is a safe method for some benign and malignant diseases, and the mortality rate of liver resection has decreased to <5% due to advances in technology, surgical techniques, and a better understanding of hepatic physiology and anatomy [1]. In particular, liver resection for colorectal cancer metastasis affects survival positively. In fact, some patients have a potentially curative treatment [2]. However, liver failure is one of the most common complications of liver resection surgery and the main cause of morbidity and mortality [3–5]. Factors predictive of liver failure include patient, surgical, and postoperative factors. Diabetes mellitus, obesity, steatohepatitis, hepatitis B and C, malnutrition, renal failure, hyperbilirubinemia, thrombocytopenia, pulmonary disease, liver cirrhosis, and age >65 years which are factors predictive of postoperative liver failure. Postoperative factors include hemorrhage and intra-abdominal infections. Operative factors include a blood loss >1200 mL during surgery, blood transfusion requirement, resection of the vena cava or other vessels, operative time >240 min, resected liver volume >50%, right lobe resection with major hepatectomy, a skeletonized hepatoduodenal ligament, and remnant liver <25% of the liver mass [4]. Portal vein embolization and two-stage hepatectomy methods are currently applied successfully to prevent postoperative liver failure [6]. However, estimated postresection liver volume is one of the most important factors limiting resection. A rapid regeneration method after hepatectomy could expand the indications for liver resection in selected patients.

The human amniotic membrane (HAM) is currently used to treat corneal wounds in patients with burns injuries and intestinal anastomosis, as it has a positive healing effect [7, 8]. The HAM also has hypoimmunogenic properties and has been used as a xenograft without tissue rejection [7, 9]. Because of this feature in many animal experiments study, human amniotic membrane was successfully used as xenograft and allograft [7, 8, 10]. In the present study, we investigated the effects of overlaying the HAM on remnant liver and on its regeneration after liver resection.

## 2. Materials and Methods

This study was performed at the Kocaeli University Experimental Animal Research Center with approval (number = 7/2 − 2013) from the Kocaeli University Institutional Ethics Committee.

### 2.1. Animals

Twenty female Wistar rats (weight range, 212–275 g; same age) were used in this study. Standard rat chow and tap water were provided to the rats. The rats were maintained under a 12-h light:dark photoperiod and normal laboratory conditions. All surgical procedures were performed in the afternoon to prevent an influence of the circadian rhythm. The rats were divided randomly into control (*n* = 10) and experimental groups (*n* = 10).

### 2.2. HAM Preparation

Written consent was obtained from pregnant women who were negative for hepatitis B and C as well as human immunodeficiency virus. The placenta was transported quickly after cesarean section to the laboratory in a sterile box with saline. A 20 mL sterile syringe was immersed in the saline and used to separate the membrane with air. The HAM was dissected while immersed in Dakin's solution for 15 min to clean off the blood and other contaminants. Mechanical cleaning was performed using saline. The HAM was incubated with saline containing 50 mg/mL penicillin, 50 mg/mL streptomycin, 100 *μ*g/mL neomycin, and 2.5 mg/mL amphotericin B for 10 min. The HAM was sectioned into 1.5- × 3-cm pieces and maintained between wet sterile cloths impregnated with saline.

### 2.3. Surgical Procedures

The rats were fasted for 6 h before surgery. Anesthesia was induced by intraperitoneal administration of 50 mg/kg ketamine (Ketalar; Parke Davis, İstanbul, Turkey) and 5 mg/kg xylazine hydrochloride (Rompun; Bayer AG, Leverkusen, Germany). The rats were provided water. The abdominal area was disinfected with 10% povidone-iodine solution, and a midline abdominal incision was made under sterile conditions. The left lateral and medial lobes of the liver were cut, and a 70% hepatectomy was performed based on the method described by Higgis [11, 12]. The abdominal incision was closed using continuous 3/0 Prolene sutures. After the hepatectomy, the 1.5- × 3-cm sections of HAM were overlaid onto the remnant liver in the experiential group. All other procedures were performed exactly the same between the two groups. Resected liver weight was recorded as the excision weight (mg). The rats were offered food and water postoperatively. Approximately 72 h later, the rats were anesthetized using the same method, and their weights (g) were measured. The abdominal and chest cavities were opened via a midline thoracoabdominal incision. Blood (2 mL) was drawn from the heart prior to excision of the remnant liver. The remnant liver weight (mg) was measured, and a specimen was maintained in 10% formaldehyde for pathological examination. The blood samples were centrifuged for 5 min at 3500 rpm, and the serum fraction was transferred to a separate tube for biochemical analyses.

### 2.4. Relative Liver Weight

Preoperative liver weight was considered to be 3.4% of the preoperative body weight and calculated [13]. Residual liver weight was calculated as the excised liver weight subtracted from the preoperative liver weight. Regenerative liver weights were calculated by subtracting the residual liver weight from the remnant liver weight during necropsy. Relative liver weight was calculated as the regenerative liver weight divided by the preoperative liver weight multiplied by 100. Relative liver weight was standardized as such [14].

### 2.5. Biochemical Analyses

Blood samples were collected from the heart, and the serum was separated by centrifugation (3000 rpm at 4°C for 15 min) and stored at −80°C for biochemical analyses. Serum total protein (T Prot) g/dL, total bilirubin (T Bil) mg/dL, direct bilirubin (D Bil) mg/dL, indirect bilirubin (I Bil) mg/dL, and albumin (Alb) g/dL levels were determined using an automated Architect C-4000 analyzer (Abbott Laboratories, Abbott Park, IL, USA). Aspartate aminotransferase (AST) IU/L, alanine aminotransferase (ALT) IU/L, and lactate dehydrogenase (LDH) IU/L levels were measured using an Architect C-8000 autoanalyzer (Abbott Laboratories) at Bozok University, School of Medicine, Central Laboratory, Yozgat, Turkey.

### 2.6. Histopathological Analyses

All specimens were transported to the pathology laboratory in 10% neutral formalin. Approximately 1-mm^2^ tissue pieces were sampled from each case. All samples were processed in an automated system (Excelsior ES; Thermo Scientific, Rockford, IL, USA), and paraffin blocks were prepared using the HistoStar embedding station (Thermo Scientific). Two tissue sections 3–6-*μ*m-thick were obtained from each case using a microtome (Shandon-Finesse ME+; Thermo Scientific). One was stained with hematoxylin and eosin (H&E) using an automated slide staining machine (Varistain Gemini; Thermo Scientific), and the other one was stained using a Ki-67 antibody (7 mL-RTU, mouse anti-human monoclonal antibody clone K2; Leica Biosystems, Danvers, MA, USA) in an automated immunohistochemical staining system (Leica-Bond Max, Leica Biosystems). The H&E- and Ki-67-stained slides were evaluated under a light microscope (BX53F; Olympus, Tokyo, Japan) by a pathologist. Nuclear staining for Ki-67 was considered as positive. The number of Ki-67-stained cells was counted in three different areas containing most of the Ki-67 positive cells using a ×100 objective with immersion oil. Then, the arithmetic mean of the number of Ki-67-positive cells was calculated. All H&E sections were evaluated for mitotic figures in 10 high power fields (×40 objective) and for histopathological changes.

### 2.7. Statistical Analyses

The SPSS for Windows 18.0 (SPSS Inc., Chicago, IL, USA) software package was used for statistical analyses. Means, standard deviations, ranges, and percentages were calculated. The Mann-Whitney *U* test was used for normally distributed data of weight loss, mitotic count, and levels of AST, LDH, total and direct bilirubin, and C-reactive protein. Ki-67 expression, relative liver weight, ALT, and total protein values were in the normal range. Student's *t*-test was used to examine these parameters. A *P* value < 0.05 was considered significant.

## 3. Results

Two rats in the control group died during the surgical procedure due to vena cava injuries. Direct bilirubin value was >7 mg/dL due to an iatrogenic bile duct obstruction in one rat in the experimental group, and the rat was excluded. Thus, nine rats were included in the experimental group and eight in the control group.

Mean preoperative body weights in the experimental and control group were 246.33 and 256.63 g, and postoperative weights were 221.11 and 221.00 g, respectively. Weight loss was calculated to be 31.38 g. No difference was detected between the groups in terms of weight loss (*P* = 0.277).

**Figure 1:**
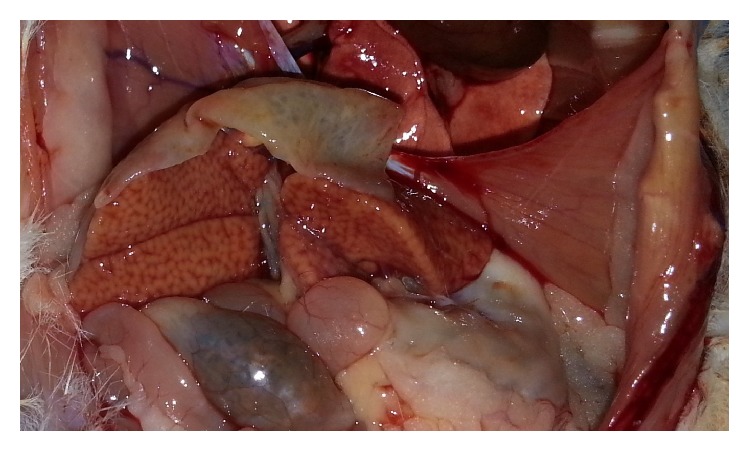
Resected liver in study group.

**Figure 2:**
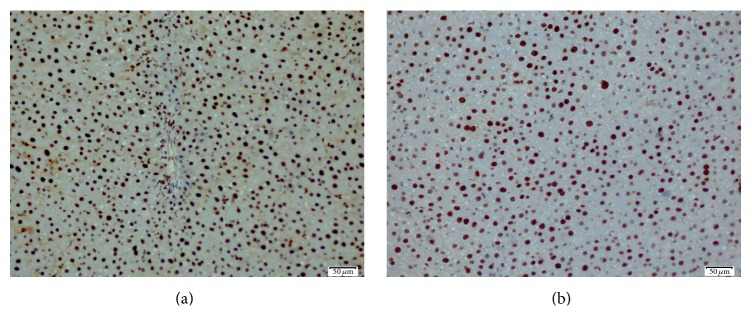
(a) Microscopic photo illustrating high Ki-67 positivity of a case of control group (Avidin-biotin-peroxidase method, ×200). (b) Microscopic photo illustrating high Ki-67 positivity of an amniotic membrane used case (Avidin-biotin-peroxidase method, ×200).

**Figure 3:**
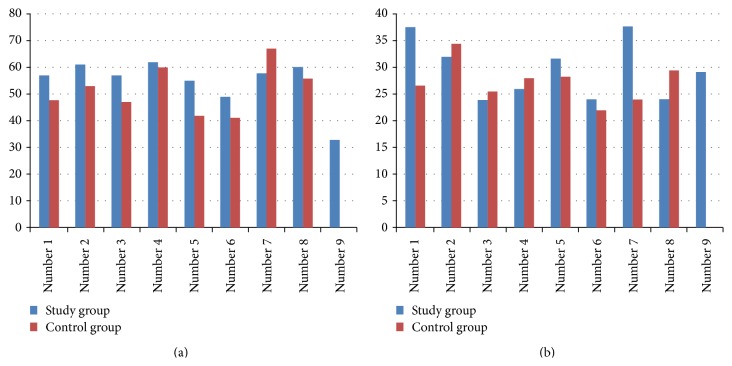
(a) Ki-67 levels. (b) Relative Liver weights (g).

**Figure 4:**
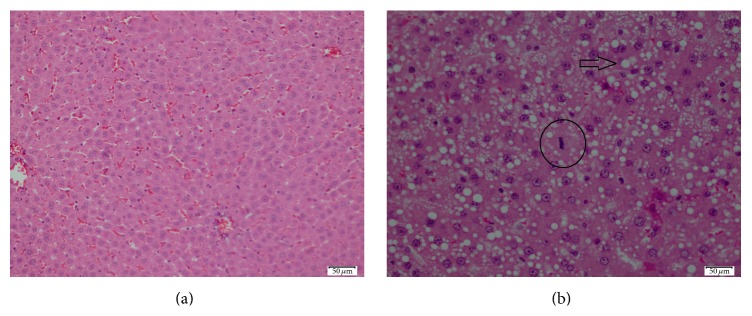
(a) The biopsy of a case of control group showing no considerable specific pathology (H&E, ×200). (b) The photomicrograph of an amniotic membrane used case showing extensive macrovesicular steatosis (*arrow*) and a mitotic figure (*circle*) (H&E, ×400).

The control group had normal appearance of the resected livers. But, in the study group, they were like fatty liver disease (Figure 1). The mean resected liver weight was 5.41 ± 0.36 g in the experimental group and 5.82 ± 0.43 g in the control group. Excision percentages were calculated according to the preoperative liver weight. Consequently, a mean of 64.83% of the liver tissue was excised in the experimental group and 66.76% in the control group (*P* = 0.103). The results of the biochemical analysis are shown in Table 1. Serum total protein and albumin levels were higher in the control group than in the experimental group (*P* < 0.01). No significant differences were observed in the AST, ALT, LDH, and total and direct bilirubin levels between the groups. The mean Ki-67 level in the postoperative remnant liver tissue was 54.67 ± 8.99 in the experimental group and 51.75 ± 9.00 in the control group (*P* = 0.515) (Figure 2). Relative liver weight tended to be higher in the experimental group (29.61 ± 5.60 g) than in the control group (27.34 ± 3.77 g) (*P* = 0.348). In contrast, the mitotic index in the remnant liver tissue was 4.22 ± 2.91 in the experimental group and 0.50 ± 1.07 in the control group (*P* < 0.01) (Table 2, Figure 3). Histopathologically, there was no specific change, tumor cell, and significant difference in inflammatory cells in the liver of study and control groups. However, macrovesicular steatosis with large and small droplets was detected in all postoperative remnant liver tissues. Steatosis comprised 70% to 95% of those tissues. Thus, severe hepatic macrovesicular steatosis was observed in residual liver tissue from the experimental group compared with the control group (Figure 4).

## 4. Discussion

The liver is capable of regenerating and can reach its previous size and function quickly. Thus, hepatic resections are often completed easily without complications, although posthepatectomy liver failure may arise in cases of massive hepatic resection [15]. The liver regeneration process in rats and mice is completed within 5–7 days after surgery [16]. The incidence rate of liver failure is reported to be 10% in patients undergoing major liver resection [17]. Posthepatectomized liver failure is a major cause of mortality and morbidity in patients undergoing liver resection [4]. According to Shoup et al., posthepatectomy liver failure results in a threefold increased risk of death in patients with a postresection volume <25% of the preoperative liver volume [18]. Methods such as portal vein embolization and two-stage hepatectomy have been used successfully to increase residual volume [6]. Techniques that enable rapid regeneration of residual liver volume can be effective for preventing posthepatectomy failure and expand resection indications.

Liver regeneration is regulated by complex mechanisms. Intercellular communication, growth factors, and cytokines, particularly hepatocyte growth factor, transforming growth factor-*α*, epidermal growth factor, tumor necrosis factor, and interleukin-6, play important roles in liver regeneration. Some studies have shown that these factors accelerate regeneration and growth of the liver mass [19, 20].

HAM is a mesenchymal stem cell source that expresses these factors [7]. HAM has the potential for cells to differentiate into hepatocytes, cardiomyocytes, chondrocytes, pancreatic B-cells, and neuronal cells [9]. Laminin is a major component of the HAM that plays an important role in cell differentiation and shaping. [9]. Jia et al. resected 70% of the rat liver and showed a positive effect on liver regeneration by placing HAM on the abdominal wall muscles [10].

We found no difference in AST, ALT, LDH, or total and direct bilirubin levels between the groups in our study. Total protein and albumin levels were significantly lower in the experimental group than in the control group. No differences in relative liver weights or Ki-67 levels were detected between the groups on postoperative day 3. Severe hepatic steatosis was observed in the experimental group compared with the control group. Based on these findings, we concluded that applying the HAM was ineffective for enhancing liver regeneration and could lead to liver cirrhosis. Previous studies used the HAM as a xenograft without tissue rejection reactions in organs such as the intestines, skin, and cornea [7, 8, 21]. The HAM is hypoimmunogenic with no HLA class II antigens. It has low levels of HLA class IA and high levels of class IB and HLA-G [9, 22]. Jia et al. showed that placing HAM in the abdominal muscles increased the rate of liver regeneration [10]. We believe that our negative results were caused by immune response mechanisms, cytokines, and cell interactions. However, the evaluation parameters were inadequate in our study. The number of mitoses was higher in the experimental group than in the control group, which was a sign of increased liver regeneration induced by the HAM. However, the increase in mitosis was not reflected by an increase in relative liver weight. We do not have mitotic results from day 1 or 7. In the literature, we found no experiments using rat amniotic membrane. But we think that using allograft may be of different results. Thus, additional studies are needed using different methods.

## 5. Conclusion

Spreading the HAM on rat liver negatively affected liver regeneration. Nevertheless, stem cell research, such as studies using the HAM, continues to provide hope for postoperative liver regeneration. Further clinical and experimental studies are needed to understand the mechanisms.

## Figures and Tables

**Table 1:** The results of the biochemical analysis.

*n*	T Prot (g/dL)	Alb (g/dL)	T bil (mg/dL)	D Bil (mg/dL)	AST (IU/L)	ALT (IU/L)	LDH (IU/L)
Study group							
1	6,4	3,1	0,1	0,1	163	76	74
2	6,3	3,2	0,2	0,1	171	96	217
3	5,9	3	0,2	0,1	234	99	349
4	5,3	2,5	0,1	0,1	241	89	212
5	6,5	3,2	0,2	0,2	221	136	144
6	6	3	0,1	0,1	162	76	580
7	5,5	2,8	0,1	0,1	433	100	196
8	5,9	2,8	0,1	0,1	192	77	625
9	6	3	0,2	0,1	159	88	176

Control group							
1	7,3	3,3	0,2	0,1	326	123	654
2	7,3	3,5	0,2	0,1	141	103	324
3	6,8	3,2	0,2	0,1	171	114	140
4	6,7	3,2	0,2	0,1	196	103	543
5	6,8	3,2	0,1	0,1	155	118	503
6	6,9	3,2	0,2	0,1	222	110	400
7	7,5	3,3	0,2	0,1	161	78	254
8	6,3	3	0,2	0,1	182	103	190

**Table 2:** Ki-67 levels and evaluation of liver weights.

*n*	Ki-67 level	Resected liver weigh (g)	Residual liver weigh (g)	Relative liver weight (g)
Study group				
1	57	4,89	2,32	37,82
2	61	5,84	3,51	31,97
3	57	4,92	2,97	23,9
4	62	5,54	3,27	25,87
5	55	5,49	3,18	31,67
6	49	5,32	2,84	24,19
7	58	5,68	2,55	37,77
8	60	5,24	2,58	23,99
9	33	5,86	3,39	29,31

Control group				
1	48	5,1	2,72	26,59
2	53	5,97	2,81	34,49
3	47	5,56	3,18	25,62
4	60	6,06	2,99	27,99
5	42	6,32	2,76	28,38
6	41	5,59	2,88	21,94
7	67	5,69	2,95	24,24
8	56	6,36	2,89	29,43
